# RETRACTED: Mehenni et al. Preparation and Characterization of Spherical Amorphous Solid Dispersion with Amphotericin B. *Pharmaceutics* 2018, *10*, 235

**DOI:** 10.3390/pharmaceutics18101283

**Published:** 2026-10-10

**Authors:** Lyes Mehenni, Malika Lahiani-Skiba, Guy Ladam, François Hallouard, Mohamed Skiba

**Affiliations:** 1 UFR of Health, Laboratory of Pharmaceutical & Biopharmaceutical Technology, Inserm U1239, UNIROUEN, Normandy University, 76183 Rouen CEDEX, France; 2 PBS (UMR 6270), CNRS, UNIROUEN, Normandy University, 27000 Evreux, France

The journal retracts the article titled “Preparation and Characterization of Spherical Amorphous Solid Dispersion with Amphotericin B” [1], cited above.

Following publication, concerns were brought to the attention of the Editorial Office regarding the integrity of an image presented in this article [1].

In accordance with the journal’s standard procedures, the Editorial Office and Editorial Board conducted an investigation that confirmed image duplication between Figure 2 of this article [1] and Figure 5 of an earlier publication [2] by a similar authorship group, despite the images being reported as representing different experimental conditions.

The authors fully cooperated with the investigation and confirmed the identified image duplication. However, the Editorial Board determined that the concerns could not be satisfactorily resolved due to insufficient supporting information and the unavailability of the underlying data, including the original SEM images.

As a result, the Editorial Board has lost confidence in the reliability of the findings reported in this article and has decided to retract the publication in accordance with MDPI’s retraction policy.

This retraction was approved by the Editor-in-Chief of the journal *Pharmaceutics*.

The authors have been informed of this retraction and have indicated their agreement with this decision.
